# Learned helplessness and social avoidance in the Wistar-Kyoto rat

**DOI:** 10.3389/fnbeh.2014.00109

**Published:** 2014-04-01

**Authors:** Hyungwoo Nam, Sarah M. Clinton, Nateka L. Jackson, Ilan A. Kerman

**Affiliations:** ^1^Department of Psychiatry and Behavioral Neurobiology, University of Alabama at BirminghamBirmingham, AL, USA; ^2^Cell, Molecular, and Developmental Biology, Graduate Biomedical Sciences Program, University of Alabama at BirminghamBirmingham, AL, USA

**Keywords:** Wistar-Kyoto (WKY), spontaneously hypertensive rat (SHR), forced swim test, elevated plus maze, social interaction, maternal separation, heart, adrenal

## Abstract

The Wistar-Kyoto (WKY) rat is an established depression model characterized by elevated anxiety- and depression-like behavior across a variety of tests. Here we further characterized specific behavioral and functional domains relevant to depression that are altered in WKY rats. Moreover, since early-life experience potently shapes emotional behavior, we also determined whether aspects of WKYs' phenotype were modifiable by early-life factors using neonatal handling or maternal separation. We first compared WKYs' behavior to that of Sprague–Dawley (SD), Wistar, and Spontaneously Hypertensive (SHR) rats in: the open field test, elevated plus maze, novelty-suppressed feeding test, a social interaction test, and the forced swim test (FST). WKYs exhibited high baseline immobility in the FST and were the only strain to show increased immobility on FST Day 2 vs. Day 1 (an indicator of learned helplessness). WKYs also showed greater social avoidance, along with enlarged adrenal glands and hearts relative to other strains. We next tested whether neonatal handling or early-life maternal separation stress influenced WKYs' behavior. Neither manipulation affected their anxiety- and depressive-like behaviors, likely due to a strong genetic underpinning of their phenotype. Our findings indicate that WKY rats are a useful model that captures specific functional domains relevant to clinical depression including: psychomotor retardation, behavioral inhibition, learned helplessness, social withdrawal, and physiological dysfunction. WKY rats appear to be resistant to early-life manipulations (i.e., neonatal handling) that are therapeutic in other strains, and may be a useful model for the development of personalized anti-depressant therapies for treatment resistant depression.

## Introduction

Depression and anxiety disorders are among the most common and burdensome health problems worldwide (Krishnan and Nestler, 2008; Nestler and Hyman, 2010). Despite pharmacological therapeutic advances, treatment-resistant forms of these psychiatric disorders still prevail (Samuels et al., 2011), and our understanding of their pathophysiology remains weak compared to other common chronic conditions (Krishnan and Nestler, 2008, 2010; Nestler and Hyman, 2010). Valid animal models are critical to elucidate mechanisms relevant to the pathophysiology of these conditions and ultimately novel therapeutic strategies (Cryan et al., 2002; Overstreet, 2012). Developing satisfactory animal models is challenging, mainly because of the complex nature of psychiatric illnesses, emotional behavior, and the brain. A popular approach is to utilize rodent models that capture behavioral endophenotypes reminiscent of human disorders in paradigms that can be readily tested and analyzed (Cryan et al., 2002; Cryan and Holmes, 2005; Nestler and Hyman, 2010; Cryan and Sweeney, 2011).

A variety of rodent models of depression and anxiety have been proposed. Many of these models include rats that exhibit either a natural susceptibility or resilience to stress, depressive- and/or anxiety-like behaviors (Armario et al., 1995; Hall et al., 2000; Shepard and Myers, 2008). Among these models, the Wistar-Kyoto (WKY) rat is a prominent and frequently used rodent model of depression (Paré, 1989; Paré and Redei, 1993; Armario et al., 1995; Pardon et al., 2002; Ferguson and Gray, 2005). The WKY rats were originally bred as a normotensive control strain for the spontaneously hypertensive rat (SHR) (Louis and Howes, 1990), but were later recognized for their susceptibility to develop stress-induced ulcers (Paré, 1989; Paré and Redei, 1993; Paré and Kluczynski, 1997; Tejani-Butt et al., 2003). Compared to other rat strains, WKYs also exhibit high levels of depression-like behavior (immobility) in the Forced Swim Test (FST) (Paré, 1989; Paré and Redei, 1993; Tejani-Butt et al., 1994, 2003; Armario et al., 1995; Marti and Armario, 1996; Lahmame et al., 1997; Paré and Kluczynski, 1997; Lopez-Rubalcava and Lucki, 2000; Rittenhouse et al., 2002), enhanced anxiety-like behavior (Paré, 1989; Paré and Redei, 1993; Paré and Kluczynski, 1997; Pardon et al., 2002; Ferguson and Gray, 2005; Shepard and Myers, 2008), and diminished activity in a novel environment (Paré, 1989; Armario et al., 1995; Marti and Armario, 1996; Paré et al., 2001; Pardon et al., 2002; Ferguson and Gray, 2005). Moreover, WKY rats exhibit altered neuroendocrine stress responses, including increased plasma corticosterone and adrenocorticotropic hormone (ACTH) levels, which resemble abnormalities that are common in depressed patients (Solberg et al., 2001; Pardon et al., 2002; Rittenhouse et al., 2002; De La Garza and Mahoney, 2004). WKY rats also display impaired gastric accommodation and visceral hypersensitivity, likely due to their exaggerated response to stress (Gunter et al., 2000; Nielsen et al., 2006). Visceral hypersensitivity is one of the main characteristics of irritable bowel syndrome (IBS), and persistence and severity of IBS symptoms is strongly related to stress responsivity (Spiller, 2004; Gibney et al., 2010; O'Mahony et al., 2010). In fact, IBS has been shown to be co-morbid with depression and anxiety (Spiller, 2004; O'Malley et al., 2010), further suggesting that the WKY rat can serve as a good animal model for studying these psychiatric diseases.

In spite of this substantial body of work, behavioral characterization of the WKY model remains incomplete. There are still additional behavioral domains relevant to clinical depression and anxiety that require further examination in WKY rats. Moreover, given the importance of early-life experience in shaping emotional behavior (Levine et al., 1957, 1967; Levine, 1962; Grota and Ader, 1969; Meaney et al., 1989; Ladd et al., 2000), it is important to determine whether WKY rats' high depressive-/anxiety-like phenotype is affected by early-life experiences. Finally, beyond these issues, there is a question of the appropriate comparison rat strain for WKY studies. As noted above, WKY rats were originally bred as a control strain for SHR rats, with both lines being derived from a common Wistar strain (Okamoto and Aoki, 1963). While the Wistar and SHR strains share a common genetic background and have been compared to the WKY numerous times, the full profile of their behavioral difference is yet to be elucidated. In addition, many published studies have described behavioral and neurochemical characteristics of the WKY rat using Sprague–Dawley (SD) rats as a control strain (Lopez-Rubalcava and Lucki, 2000; Ferguson and Cada, 2004; McAuley et al., 2009); SD rats have a distinct genetic background from WKYs and it is unclear if prior reported difference could possibly arise due to heterogeneous genetic backgrounds.

The first aim of the present study was to more fully characterize WKY rats' behavioral profile using a test battery that included assessments not previously reported in these animals, including a time-course analysis for the FST, a test of behavioral despair, and a social interaction test comparing experimental animals' interactions with a novel object, a male or a female interaction partner. We compared WKY rats' performance in these tasks, as well as classic tests of behavioral reactivity to anxiogenic environments and appetitive cues, to that of Wistar, SHR, and SD rats. We also contrasted body weight, heart weight, and adrenal gland weight across the four strains to explore potential physical abnormalities that may be associated with WKYs' depression-like phenotype.

The second part of the study investigated whether WKYs' behavioral profile is modifiable by early-life experience. We therefore utilized two different experimental paradigms, separating WKY rat pups from their mothers for either a short period each day [15 min, Neonatal Handling (MS15)], or a prolonged period each day [180 min, Maternal Separation (MS180)] during the first 2 weeks of life. Previous studies demonstrated the protective effects of MS15 on rat behavioral and endocrine stress responses (Levine et al., 1957; Meaney et al., 1989; Viau et al., 1993; Bhatnagar and Meaney, 1995), while MS180 typically elicits several negative behavioral, neuroendocrine, and neurobiological effects in other rat strains (Ladd et al., 2000; Huot et al., 2001; Holmes et al., 2005; Aisa et al., 2009; Roth et al., 2009). The impact of MS180 and especially MS15 on WKY rats is not well-known, so this experiment tested the hypothesis that: (a) exposing WKY rats to MS15 would reduce their depression- and or anxiety-like behavior, while (b) exposing them to MS180 would exacerbate their high depression/anxiety-like phenotype.

## Methods

### Animals

The first experiment used adult male rats (6–7 weeks old) of four different strains (Wistar, WKY, SHR, and SD; *n* = 12 per strain) obtained from Charles River Laboratories (Kingston, NY). Rats were group-housed 3 per cage within strain and acclimated to our animal facility for 7 days before the start of the behavior testing. The second experiment used 16 primiparous timed-pregnant WKY females that were delivered to our animal facility at gestational day 15. Dams and litters were handled as described below for the MS15/MS180 protocol. At weaning [postnatal day (P)21] only male offspring were kept for later experiments. At this time, weaned male offspring were housed 3 per cage (within experimental groups—control/MS15/MS180) through adulthood when they embarked on a behavioral test battery. The animal housing facility was maintained on a 12/12 h light-dark cycle with the lights on at 06:00. All testing procedures were approved by the University of Alabama at Birmingham Institutional Animal Care and Use Committee.

### Behavior test battery

All behavior tests, except for the sucrose preference test, were conducted under dim light (30 lux) conditions between 8:30 and 11:30 am. Animals were habituated to the testing room by placing home-cages in the test room overnight prior to testing; this habituation process was used for all tests except the sucrose preference test (SPT) and the FST. The same animals were assessed across all behavioral tests with at least 2 days of resting in between tests.

#### Sucrose preference test (SPT)

Rats were group-housed three per cage, and all animals within each cage were tested together (*n* = 4 per strain). The animals were initially presented with two bottles filled with tap water, which were weighed 24-h later to assess baseline home-cage water consumption. One of the water bottles was then filled with 1% sucrose solution, and rats were allowed to drink freely for the next 24-h. After this time, the water and sucrose bottles were removed and weighed again. Sucrose preference was calculated from data obtained on the second day of the test as follows: (volume of sucrose solution consumed/total volume of liquid consumed) × 100%.

#### Open field test (OFT)

The OFT was conducted in a 100 × 100 × 50 cm black Plexiglas box with a black floor. At the beginning of the test, a rat was placed in a corner of the box and was permitted to explore the apparatus for 5 min. The latency to enter the center of the OF, the amount of time spent and distance traveled in the center, periphery, and corners of the apparatus were quantified utilizing Ethovision® XT 8.0 videotracking software (Noldus, Wageningen, The Netherlands) set up with a digital video camera. A trained observer that was blinded to experimental groups manually assessed grooming and rearing behavior using a computerized system provided in the software.

#### Elevated plus maze (EPM)

The EPM was constructed of black Plexiglas with four elevated arms (70 cm from the floor, 45 cm long, 12 cm wide) arranged in a cross. Two opposite arms were enclosed by 45-cm-high walls, while the other two arms were open. At the start of each test, a rat was placed in the center square of the EPM facing a closed arm, then allowed to explore freely for 5 min. Using the Ethovision® XT 8.0 system, the following behaviors were quantified: latency to enter the open arms, amount of time spent and distance traveled in the open arms, closed arms, and center square.

#### Novelty-suppressed feeding (NSF)

Rats were food-deprived overnight and then were tested in a novel environment in the Noldus PhenoTyper® Boxes (45 × 45 × 60 cm clear Plexiglas chambers described above). At the start of the test session, a single food pellet of standard rat chow was placed in the center of the chamber; a rat was then placed in the periphery of the box and permitted to freely explore for 10-min. Latencies to sniff, touch, and eat the food pellet were measured by a trained observer blinded to experimental groups. The animals' movement was assessed by Ethovision® XT 8.0 software. Food pellet weight was manually measured before and after each session. As a control, home-cage food consumption was assessed by weighing each cage's food at the beginning and end of a 24-h period.

#### Forced swim test (FST)

Testing was performed as described by Cryan et al. (2005) in clear Plexiglas cylinders (40 cm high × 40 cm diameter) containing 30-cm deep water (25°C). On day 1, rats were placed (one rat/cylinder) in the water for 15-min; 24 h later the rats were returned to the water-filled cylinder and tested for 5-min (day 2). Water was changed after every swim session so that every rat was swimming in clean water. Each rat's FST behavior was digitally recorded, and immobility was scored by the Ethovision® XT 8.0. We focused on the immobility measure since it is classically considered an indicator of behavioral despair and depressive-like behavior (Porsolt et al., 1977), and it can be clearly defined and easily distinguishable from active coping measures such as swimming and climbing, which are sometimes difficult to reliably distinguish across experimental observers (Porsolt et al., 1977; Cryan et al., 2005; Muigg et al., 2007).

#### Social interaction

The social interaction test was comprised of three 10-min sessions where experimental rats encountered: (1) an empty test box during the first test day; (2) a box containing a novel male rat during the second test day; and (3) a box containing a novel female rat during the third test day. Testing was conducted within a multi-purpose Noldus PhenoTyper® Cage (a 45 × 45 × 60 cm clear Plexiglas chamber outfitted with a camera). An interaction box (a 10 × 10 × 8 cm clear Plexiglas box with breathing holes) was placed in the corner of each cage; it was used to house the novel stimulus male or female rat that the experimental animal could interact with during a test session. These stimulus rats were of the same strain, and younger (P40–45) and smaller (140–210 g) than the test animals (P70–80). Since the duration of the test was 10 min., the stimulus rats were exposed to the interaction box for increasing periods of time on the 3 training days: 5, 10, and 15 min., to habituate to its exposure. On the first social interaction test day, experimental rats were individually placed in a PhenoTyper® cage containing an empty interaction box. On the second and third social interaction test days, the interaction box contained either a novel male (day 2) or novel female (day 3) stimulus rat. Each stimulus rat was used for only a single test trial per day. In order to assess the experimental animals' level of social interaction, we defined specific zones in the Phenotype chamber as either interaction or avoidance zones. Thus, an approximately 2-cm-wide zone around the interaction box was designated as the interaction zone and a 10 × 10 cm zone in the corner diagonal from the interaction box was considered the avoidance zone. Time spent within each zone and number of visits to each zone during the 3 test sessions was quantified with Ethovision® XT 8.0 system.

### Tissue and blood collection

One week after the behavior test battery for the first experiment (comparing WKY, Wistar, SD, and SHR rats), animals were sacrificed by decapitation and trunk blood was collected. Hearts and adrenal glands were dissected and kept on ice prior to weighing within 2–3 h of collection. Whole blood was allowed to clot and centrifuged at 4000 rpm (3220 × g) for 20 min at 4°C to collect the serum, which was stored at −80°C.

### Corticosterone measurement

Serum samples were diluted and circulating corticosterone levels were assayed using a commercially-available ELISA kit (Corticosterone EIA Kit, ADI-900-097, Enzo Life Sciences) according to manufacturer instructions. According to the manufacturer, the sensitivity of the assay was 26.99 pg/ml, with the intra-assay coefficient of variation less than 8.4% and inter-assay coefficient of variation less than 13.1%. Light absorbance was read with a multi-mode plate reader (Synergy HT, BioTek Instruments, Inc.) at 405 nm. Data were analyzed using 4-point sigmoidal algorithm provided in GraphPad Prism 6.0 software (GraphPad Software, La Jolla, CA, USA).

### Early-life manipulations: neonatal handling (MS15) or prolonged maternal separation (MS180)

For the second experiment, we exposed WKY pups to either a positive or negative early-life experience: daily neonatal handling (MS15) or maternal separation (MS180), respectively, for the first two postnatal weeks. Timed-pregnant WKY females (*n* = 16) arrived at our animal facility on gestational day 15 and remained singly housed until giving birth. All litters were born on the same day, and all pups within a litter were exposed to either MS15, MS180, or were part of non-separated control litters. At birth (P0), litters were randomly assigned to experimental groups: (a) neonatal handling where pups were maternally-separated daily P1–14 for 15 min (MS15); (b) prolonged maternal separation where pups were maternally-separated daily P1–14 for 180 min (MS180); or (c) non-separated control (*n* = 5–6 litters per condition).

The following day (P1) we initiated a separation procedure modeled after Plotsky and Meaney (Plotsky and Meaney, 1993) and Card et al (Card et al., 2005). Non-separated control litters remained with their dam continuously except for once weekly cage changes. Beginning on P1 (continuing through P14), MS15 and MS180 litters were separated from their dam for 15 and 180 min, respectively. An entire litter was moved with a handful of home bedding to a small cage on a heating pad. The temperature of the nest was around 37°C when the litter was transferred to the cage over the heating pad. The separation cages were placed in a separate room from the home cage where the dam remained. Littermates remained in close contact with one another throughout the separation period; at the conclusion of the separation period, pups and bedding were returned to the home cage. Pups were weaned and culled on P21, and only males (*n* = 15 per group) were randomly chosen for subsequent behavioral tests performed in adulthood (75+ days) using the behavioral test battery described above [section Open Field Test (OFT)–Social Interaction].

### Statistical analysis

Data were analyzed using SPSS 21 (IBM Corp.) and GraphPad Prism 6. One-Way analysis of variance (ANOVA) followed by Bonferroni multiple comparison *post-hoc* test were used to determine strain effects across all experiments. The FST and social interaction results were analyzed using Two-Way repeated measures ANOVA with Bonferroni or Tukey's multiple comparisons to determine effects of: strain, 5-min time bins/partners, and interaction between the factors. Additionally, two-tailed *t*-test was also used in the FST analysis for day 1 vs. day 2 comparison. All subjects (*n* = 12 per strain) were used in statistical analysis throughout the behavioral tests, with the exception of the SPT which was analyzed by cages instead of individual animals, giving the *n* = 4 per strain.

## Results

### Anxiety- and depressive-like behavior in WKY, Wistar, SD, and SHR strains

#### Social interaction

**Figure 1:**
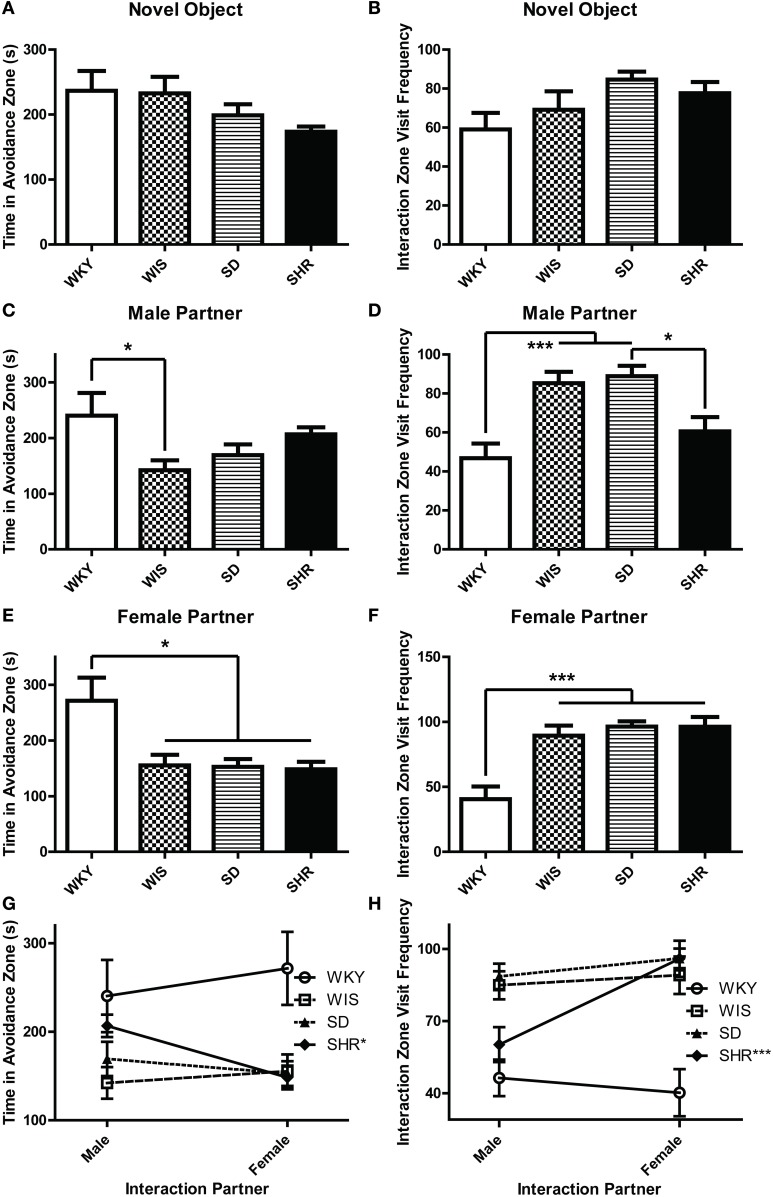
**WKY rats exhibit social interaction deficits relative to other rat strains**. Social interaction behavior was examined in adult male Wistar Kyoto (WKY), Wistar (WIS), Sprague–Dawley (SD), and Spontaneously Hypertensive (SHR) rats. During the three social interaction test sessions, experimental animals encountered a novel object (test day 1), a novel male stimulus rat (test day 2), or a novel female stimulus rat (test day 3). During the first session, all strains spent similar amounts of time in an avoidance zone farthest away from the novel object **(A)**; all of the rats also spent similar lengths of time in a designated interaction zone that was within 2-cm of the novel object **(B)**. During the second test session when animals encountered a novel male stimulus rat, WKY rats spent more time in the avoidance zone compared to other strains **(C)**, but less time in the interaction zone **(D)**. Likewise, during the third session when animals encountered a female stimulus rat, WKY rats again spent more time in the avoidance zone compared to other strains **(E)** and less time in the interaction zone **(F)**. Only the SHR showed significant preference for female than male partner rats, spending less time in the avoidance zone **(G)** and interacting more frequently **(H)** when with females. ^***^p < 0.001; ^*^p < 0.05 (male vs. female was compared in panels **G,H**).

During the three social interaction test sessions, experimental animals encountered either a novel object (the empty interaction box; Day 1), a novel male stimulus rat (Day 2), or a novel female stimulus rat (Day 3). On the first test day, all strains spent similar amounts of time in the avoidance zone [Figure 1A; effect of strain: *F*_(3, 44)_ = 1.854, *p* = 0.151] and interaction zone [data not shown; effect of strain *F*_(3, 44)_ = 0.185, *p* = 0.906]. Likewise, there was no difference in number of visits to the interaction zone [Figure 1B; effect of strain: *F*_(3, 44)_ = 2.313, *p* = 0.089]. On test day 2 when experimental animals encountered a novel male stimulus rat, WKY rats spent more time in the avoidance zone compared to the other strains [Figure 1C; main effect of strain: *F*_(3, 44)_ = 2.919, *p* = 0.044; *post-hoc p* < 0.05 for WKY vs. Wistar], and visited the interaction zone the less often [Figure 1D; main effect of strain: *F*_(3, 44)_ = 9.424, *p* < 0.001; *post-hoc p* < 0.001 for WKY vs. Wistar and SD]. Similarly, on test day 3 when the experimental animals encountered a female stimulus rat, WKY rats again spent more time in the avoidance zone [Figure 1E; main effect of strain: *F*_(3, 44)_ = 5.863, *p* = 0.002, *post-hoc p* < 0.011; WKY vs. all strains] and visited the interaction zone the less frequently [Figure 1F; main effect of strain: *F*_(3, 44)_ = 12.642, *p* < 0.001; *post-hoc p* < 0.001, WKY vs. all strains] compared to the other strains.

The SHR showed significantly less interaction with male partners compared to SDs (Figure 1D; *post-hoc p* < 0.05) but not with the female partners. So we also compared day 2 vs. day 3 data to assess a possible male vs. female stimulus rat preference. Here we used a Two-Way ANOVA, using the sex of stimulus rat and experimental rat strain as independent factors. This analysis revealed no effect of stimulus rat sex, but a main effect of strain [*F*_(3, 33)_ > 4.855, *p* < 0.007] and a significant stimulus rat sex × strain interaction in both the time spent in the avoidance zone [Figure 1G, *F*_(3, 33)_ = 3.427, *p* = 0.028] and the frequency to visit the interaction zone [Figure 1H, *F*_(3, 33)_ = 4.682, *p* = 0.008]. The SHR was the only strain that exhibited differential preference depending on the sex of the partners, showing less interaction with male than female partners (*post-hoc p* < 0.001, male vs. female) and more avoidance from male than female partners (*post-hoc p* < 0.05, male vs. female).

#### FST

**Figure 2:**
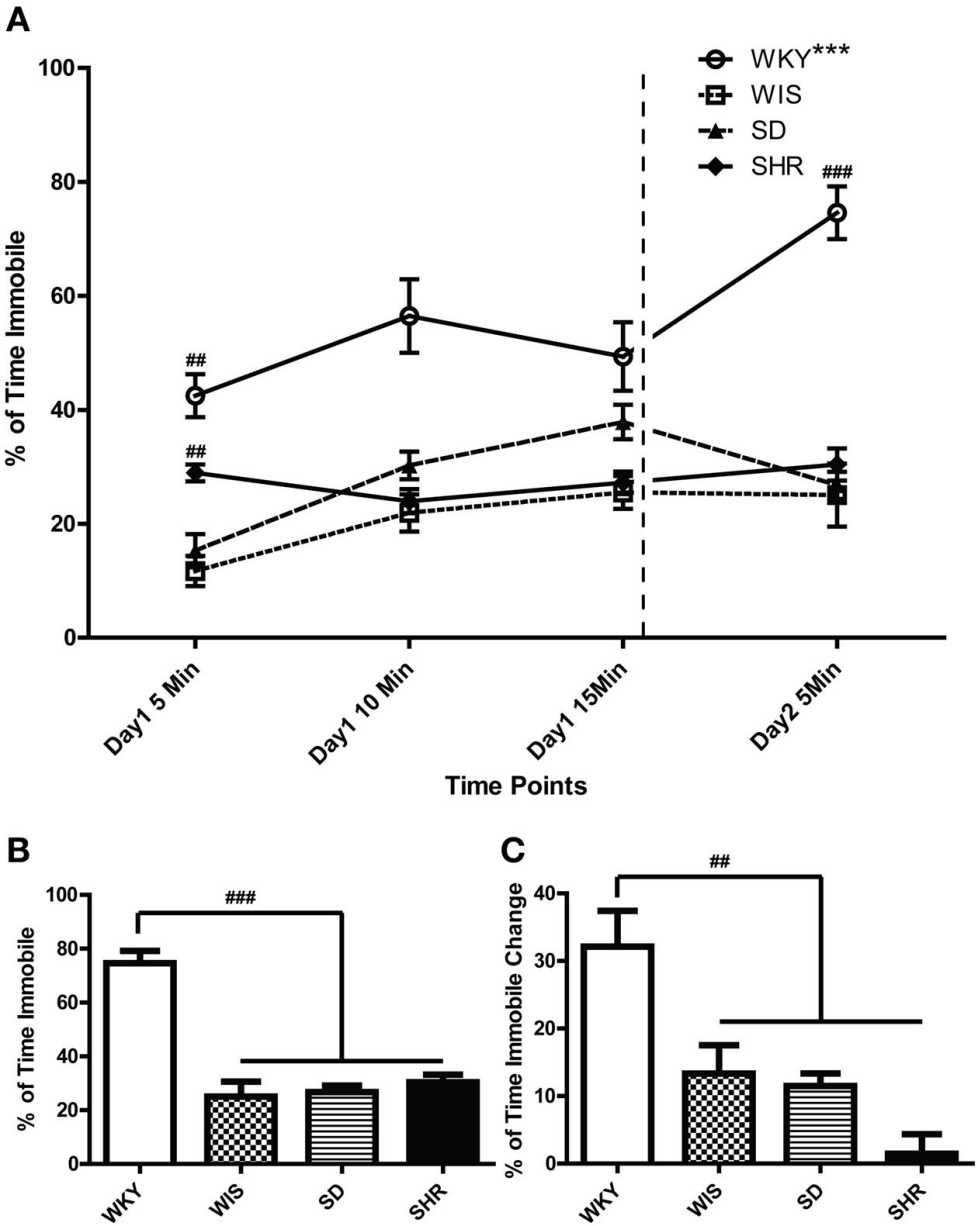
**Distinct immobility profile of WKY vs. other strains in the forced swim test**. Depressive-like behavior was assessed in adult male Wistar Kyoto (WKY), Wistar (WIS), Sprague–Dawley (SD), and Spontaneously Hypertensive (SHR) rats using a standard 2-day Porsolt forced swim test. The first day involved a 15-min swim session; 24 h later, rats were subjected to a second 5-min swim session. Percent time spent immobile was averaged over 5-min bins during the 2 days of the test **(A)**. Average percent immobility on Day 2 vs. average on Day 1 was significantly greater in WKY rats, but not in the other strains; ^***^p < 0.001 for time effect (Day 1 vs. Day 2; **A**). Day 1 immobility scores were significantly different among strains; ^##^*p* < 0.01 for strain effect (vs. all the other strains; **A**). WKY rats were significantly more immobile than the other rat strains on Day 2 (**A,B**; ^###^*p* < 0.001), and the magnitude of change in immobility during the first 5 min. bin on Day 1–2 was significantly greater in WKY rats (**C**; ^##^*p* < 0.01).

As noted above, the FST is a 2-day test that involves a 15-min swim on day 1 followed by a second 5-min swim test 24 h later. In the present study, we recorded and analyzed immobility over both test days. In the FST day 2, WKY rats were more immobile compared to the other three strains [Figures 2A,B; main effect of strain: *F*_(3, 44)_ = 33.973, *p* < 0.001; *post-hoc p* < 0.001 for WKY vs. each of the other strains]. There was no significant immobility difference among the other strains. In Figure 2A, the percent time spent immobile during the first, middle, and last 5 min of the day 1 as well as time spent immobile during the 5-min day 2 swim session are displayed. This presentation enabled us to examine how rats from each strain changed their immobility behavior over time during the day 1 session as well as how immobility changed from the first to the last swim session (Figure 2C). Statistical analysis revealed significant effects of time [*F*_(3, 33)_ = 12.02, *p* < 0.001] and strain [*F*_(3, 33)_ = 41.32, *p* < 0.001] as well as a significant time × strain interaction [*F*_(9, 99)_ = 7.570, *p* < 0.001]. *Post-hoc* tests showed that WKY rats were more immobile than any other strain across all time points (*p* < 0.05 for WKY vs. each of the other strains). Moreover, WKY was the only strain that increased its immobility from day 1 to day 2 (*p* < 0.001 for WKY day 1 immobility vs. day 2 immobility); the other three strains showed similar immobility levels across test days (Figure 2A).

#### OFT

**Figure 3:**
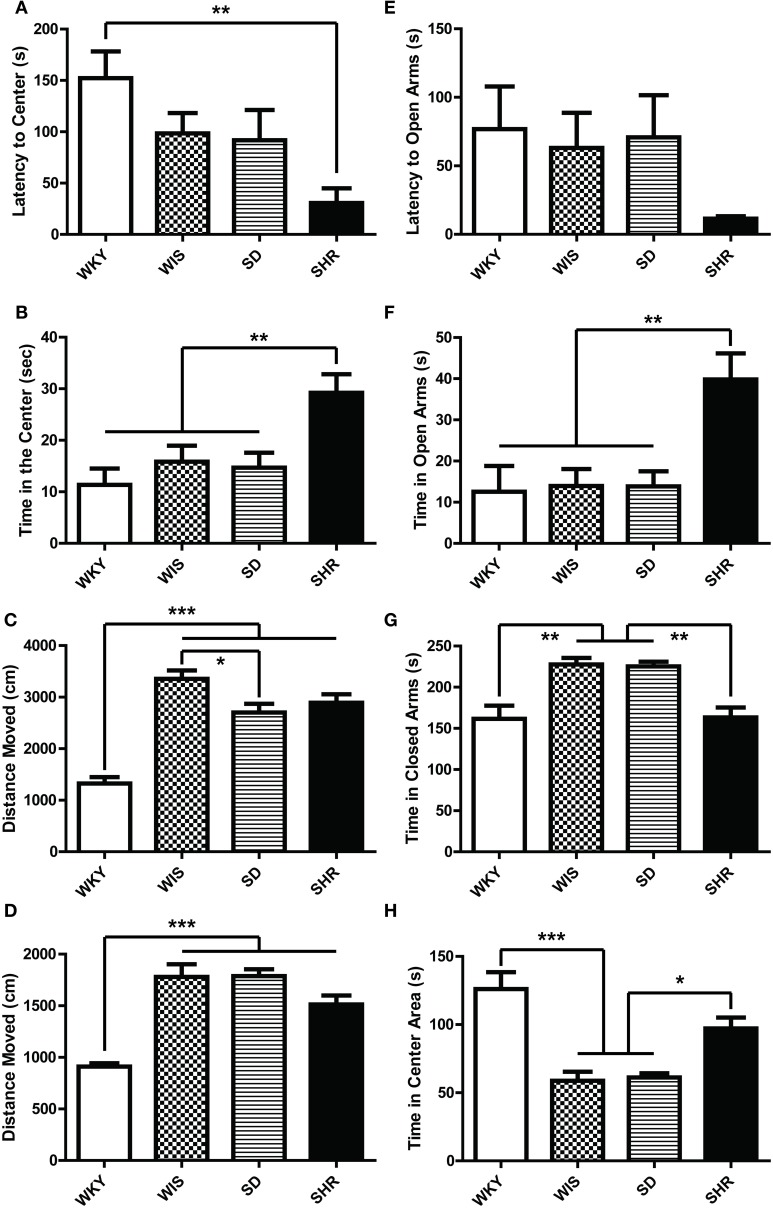
**Anxiety-like behavior of four rat strains in the open field test and elevated plus maze**. Adult male rats from four strains—Wistar Kyoto (WKY), Wistar (WIS), Sprague–Dawley (SD), and Spontaneously Hypertensive Rat (SHR)—were compared in two tests of rodent anxiety: the open field **(A–C)** and the elevated plus maze **(D–H)**. SHR showed the least anxiety with shortest latency to visit the center of the OF **(A)**, and longer time spent there **(B)** compared to all other strains. WKY rats, on the other hand, exhibited the least locomotion **(C)**. In the elevated plus maze, SHR again showed the least anxiety-like behavior relative to other strains, spending the greatest amount of time in the open arms **(F)**. SHR and WKY rats spent less time in closed arms than SD and Wistar rats **(G)**. WKY rats and SHRs also spent more time in the center square (between closed arms and open arms) compared to Wistar and SD rats **(H)**. As in the open field, WKYs showed the least locomotion in the elevated plus maze **(D)**, and there was no significant strain difference in latency to enter the open arms **(E)**. ^***^p < 0.001; ^**^p < 0.01; ^*^p < 0.05.

WKY rats showed higher latency to initially enter the center of the OF compared to SHRs [Figure 3A; main effect of strain: *F*_(3, 44)_ = 4.626, *p* = 0.007; *post-hoc p* = 0.003 for WKY vs. SHR]. SHRs spent the most time in the center of the OF compared to the other rat strains [Figure 3B; main effect of strain: *F*_(3, 44)_ = 6.034, *p* = 0.002; *post-hoc p* < 0.03 for SHR vs. each of the other strains]. There was no difference among WKY, Wistar, and SD rats on center time, and no strain difference for time spent in the OF periphery or corners (data not shown). WKYs were the least active strain, showing the shortest distance moved [Figure 3C; main effect of strain: *F*_(3, 44)_ = 31.881, *p* < 0.001; *post-hoc p* < 0.001 for WKY vs. each of the other strains]. Wistar rats were the most active strain, traversing greater distance (Figure 3C; *post-hoc p* < 0.027) than the SD and the WKY rats. WKYs exhibited less rearing in the OF compared to the other strains (data not shown; main effect of strain on rearing frequency [*F*_(3, 44)_ = 21.589, *p* < 0.001] and duration [*F*_(3, 44)_ = 20.895, *p* < 0.001]; *post-hoc p* < 0.001 for WKY vs. each of the other strains). All four rat strains showed similar amounts of grooming behavior during the OFT (data not shown).

#### EPM

SHRs spent the most time in the open arms of the EPM relative to the other three strains [Figure 3F; main effect of strain: *F*_(3, 44)_ = 6.359, *p* = 0.001; *post-hoc p* < 0.007 for SHR vs. each of the other strains]. Likewise, SHR made more frequent open arm visits compared to the other strains [data not shown; main effect of strain: *F*_(3, 44)_ = 3.731, *p* = 0.018; *post-hoc p* = 0.023 for SHR vs. SD; *p* = 0.10 for SHR vs. Wistar or WKY]. Both SHR and WKY rats spent less time in closed arms of the EPM relative to SD and Wistar rats [Figure 3G; main effect of strain *F*_(3, 44)_ = 11.040, *p* < 0.001; *post-hoc p* < 0.002 for SHR compared to SD and Wistar; *p* < 0.01 for WKY vs. SD and Wistar]. This occurred because WKY rats and SHRs spent more time in the center square (between closed arms and open arms) compared to Wistar and SD rats [Figure 3H; *F*_(3, 44)_ = 15.292, *p* < 0.001; *post-hoc p* < 0.02 for SHR or WKY vs. Wistar and SD]. All strains showed similar latency to enter the open arms (Figure 3E; no effect of strain). Finally, WKY rats were the least active of the strains tested, moving a shorter distance in the EPM [Figure 3D; main effect of strain: *F*_(3, 44)_ = 23.926, *p* < 0.001; *post-hoc p* < 0.001 for WKY vs. each of the other strains].

#### NSF

**Figure 4:**
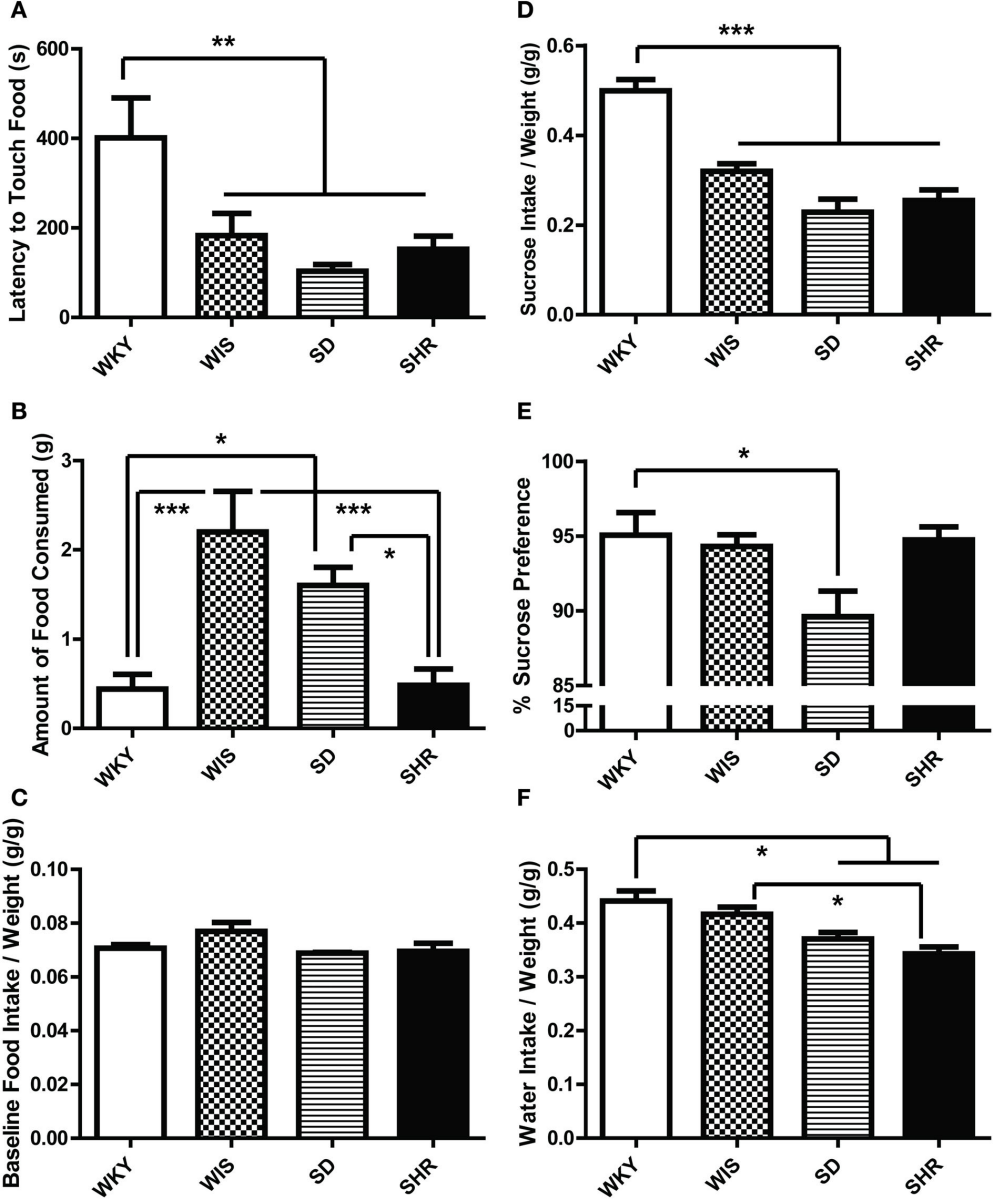
**Ingestive behavior in the novelty-suppressed feeding test and sucrose preference test**. In the novelty-suppressed feeding **(A–C)**, WKY rats showed the greatest latency to initially touch the food pellet **(A)** in the 10-min test. While the SHR showed a shorter latency to touch the food compared to WKY **(A)**, they consumed comparably low amounts of food which were significantly less than SDs and Wistars **(B)**. There was no difference in the baseline food intake measured in their home cages **(C)**. During the sucrose preference test **(D–F)**, WKYs consumed more sucrose solution than any other strains **(D)**. WKYs preferred the sucrose solution significantly only compared to SDs **(E)** because WKYs consumed more water per weight in the home-cage compared to SDs and SHRs **(F)**. Nevertheless, all four rat strains showed high preference for the sucrose solution over water **(E)**. ^***^p < 0.001; ^**^p < 0.01; ^*^p < 0.05.

Compared to the other strains, WKY rats exhibited longer latency to initially touch the food in the novel test environment [Figure 4A; main effect of strain: *F*_(3, 44)_ = 6.033, *p* = 0.002; *post-hoc p* < 0.05 for WKY vs. all other strains]. WKY rats also showed a longer latency to eat the food compared to SD rats [data not shown; main effect of strain: *F*_(3, 44)_ = 4.833, *p* = 0.005; *post-hoc p* = 0.01 for WKY vs. SD] and made fewer food pellet touches [data not shown; main effect of strain: *F*_(3, 44)_ = 5.063. *p* = 0.004; *post-hoc p* = 0.004 for WKY vs. SD]. WKY rats consumed less food than Wistar [Figure 4B; main effect of strain *F*_(3, 44)_ = 9.613, *p* < 0.001; *post-hoc p* < 0.001] and SD rats (*post-hoc p* < 0.05). Interestingly, while SHRs showed shorter latency to explore the food than the WKY rats (Figure 4A), the two strains (SHR and WKY) actually displayed similar eating behavior in terms of latency to begin ingesting the food pellet and the amount of food consumed (Figure 4B). The amount of food intake in their respective home cages were not significantly different (Figure 4C).

#### SPT

There was a strain effect in the baseline home-cage water consumption tested prior to the SPT. WKYs consumed more water per weight (g/g) compared to SDs and SHRs [Figure 4F; main effect of strain *F*_(3, 12)_ = 9.163, *p* = 0.002; *post-hoc p* < 0.032 for WKY vs. SD, WKY vs. SHR, and WIS vs. SHR]. When given access to a 1% sucrose solution, all four rat strains increased their liquid consumption due to their sucrose intake (data not shown). The strain effect was significant for the total sucrose solution intake [Figure 4D; main effect of strain *F*_(3, 12)_ = 25.902, *p* < 0.001; *post-hoc p* < 0.002 for WKY vs. all the other strains] and percent sucrose preference [Figure 4E; main effect of strain *F*_(3, 12)_ = 3.996, *p* = 0.035; *post-hoc p* = 0.047 for WKY vs. SD], although all four rat strains showed high preference for the sucrose solution over water.

### Body weight and organ weight in WKY, Wistar, SD, and SHR strains

During the first experiment comparing WKY, Wistar, SD, and SHR strains, all rats were weighed prior to the start of the behavioral test battery and just before sacrifice at the conclusion of the study. Percent body weight increase between these two time points was calculated for each group. The percent increase of animals' weight was the highest in the Wistar group compared to the other strains [Table 1; main effect of strain: *F*_(3, 44)_ = 45.914, *p* < 0.001; *post-hoc p* < 0.001 Wistar vs. each of the other strains]. The SHRs gained the least weight compared to all other strains (*post-hoc p* < 0.001 SHR vs. each of the other strains). Weight gain percentages of the WKY and the SD were comparable to each other (*post-hoc p* = 1.00).

**Table 1:** **Body and organ weights and corticosterone levels**.

**Measure (unit)**	**Wistar-Kyoto**	**Wistar**	**Sprague–Dawley**	**SHR**
Body weight (bw; g)	254.9±3.8^a^	376.0±9.3^b^	350.7±7.7^b^	268.8±3.9^a^
Body weight increase (%)	59.1±0.8^b^	73.9±3.8^c^	58.6±2.2^b^	35.6±1.3^a^
Adrenal weight (mg/100 g bw)	22.6±1.6^b^	15.7±1.5^a^	15.2±0.7^a^	14.1±0.5^a^
Heart weight (mg/100 g bw)	383.4±6.2^c^	312.9±5.4^a^	349.6±5.9^b^	422.2±11.5^d^
Corticostrone (ng/ml)	49.1±6.6^a^	62.6±6.2^a,b^	46.9±3.5^a^	84.5±9.2^b^

*Organ weights were corrected for body weight. Values represent: mean ± s.e.m. in the respective units indicated for each of 4 strains of rat: Wistar-Kyoto, Wistar, Sprague–Dawley, and SHR. Groups were compared by One-Way ANOVA; different letters indicate significant differences (*p* < 0.05)*.

The weights of heart and adrenal gland were normalized by individual body weights. Normalized heart weights differed across the strains [main effect of strain: *F*_(3, 44)_ = 37.171, *p* < 0.001; *post-hoc p* < 0.02 for each strain compared to the other]. SHRs had the largest hearts followed by the WKY, which had the second largest hearts, and Wistar, which had the smallest. WKY rats had the heaviest adrenal gland [main effect of strain: *F*_(3, 44)_ = 10.325, *p* < 0.001; *post-hoc p* < 0.001 for WKY vs. each of the other strains].

### Corticosterone levels in WKY, Wistar, SD, and SHR strains

We collected trunk blood at the conclusion or the behavioral studies in WKY, Wistar, SD, and SHR strains to evaluate baseline corticosterone levels. SHR showed higher basal corticosterone levels compared to WKY and SD (but not Wistar) rats [Table 1; main effect of strain: *F*_(3, 44)_ = 6.791, *p* < 0.001, *post-hoc p* < 0.05 for SHR vs. WKY and SD. WKY, SD, and Wistar rats showed similar basal corticosterone levels].

### Early-life experience does not alter anxiety- and depression-like behavior in WKY rats

Since the WKY rat is a well-established animal model of depression, we were interested to determine whether a positive early-life experience (neonatal handling; MS15) or early-life stressor (maternal separation; MS180) could either reduce or increase their anxiety- and depression-like behavior. Thus, in a second experiment, WKY rats were exposed to MS15 or MS180 during the first two postnatal weeks. Adult offspring were later evaluated in the behavioral test battery described for the first experiment.

#### Social interaction

**Figure 5:**
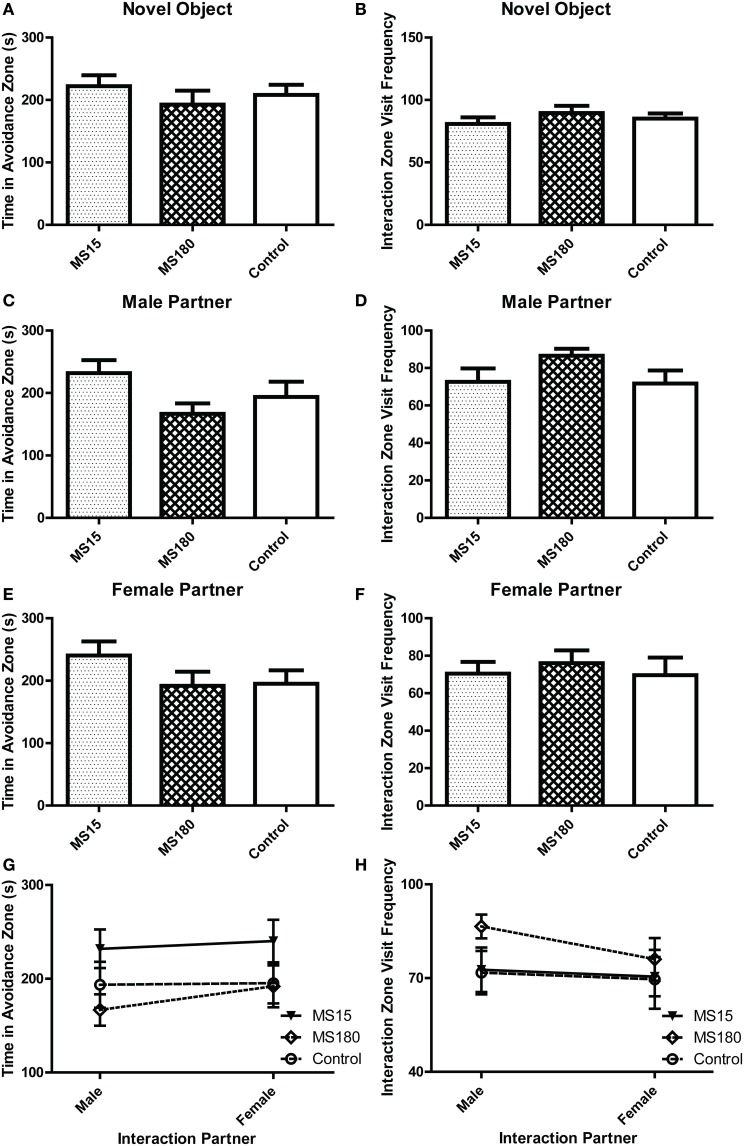
**Early-life experience does not alter WKY social interaction behavior**. Social interaction behavior was examined in adult male WKY control rats as well as WKYs that were separated from their dams for 15 min (MS15) or 180 min (MS180) per day during the first 2 weeks of life. During the three test sessions, animals encountered a novel object (test day 1), a novel male stimulus rat (test day 2), or a novel female stimulus rat (test day 3). All three groups spent similar time in the avoidance zone and make a similar number of visits to the interaction zone during the novel object exposure session **(A,B)**, the novel male stimulus rat exposure session **(C,D)**, and the novel female stimulus rat exposure session **(E,F)**. Sex preference was also not significantly different among experimental groups **(G,H)**.

During the Social Interaction test, we evaluated time spent in the avoidance zone and number of visits to the interaction zone when experimental animals were exposed to either a novel object (Figures 5A,B), a novel male stimulus rat (Figures 5C,D), or a novel female stimulus rat (Figures 5E,F). As with the other behavioral tests, there were no group differences between MS15, MS180, and control WKY rats in any of these measures. Comparing data between male vs. female partner interactions revealed no effect of partners in the time spent in the avoidance zone (Figure 5G) or the frequency of visiting the interaction zone (Figure 5H).

#### FST

**Figure 6:**
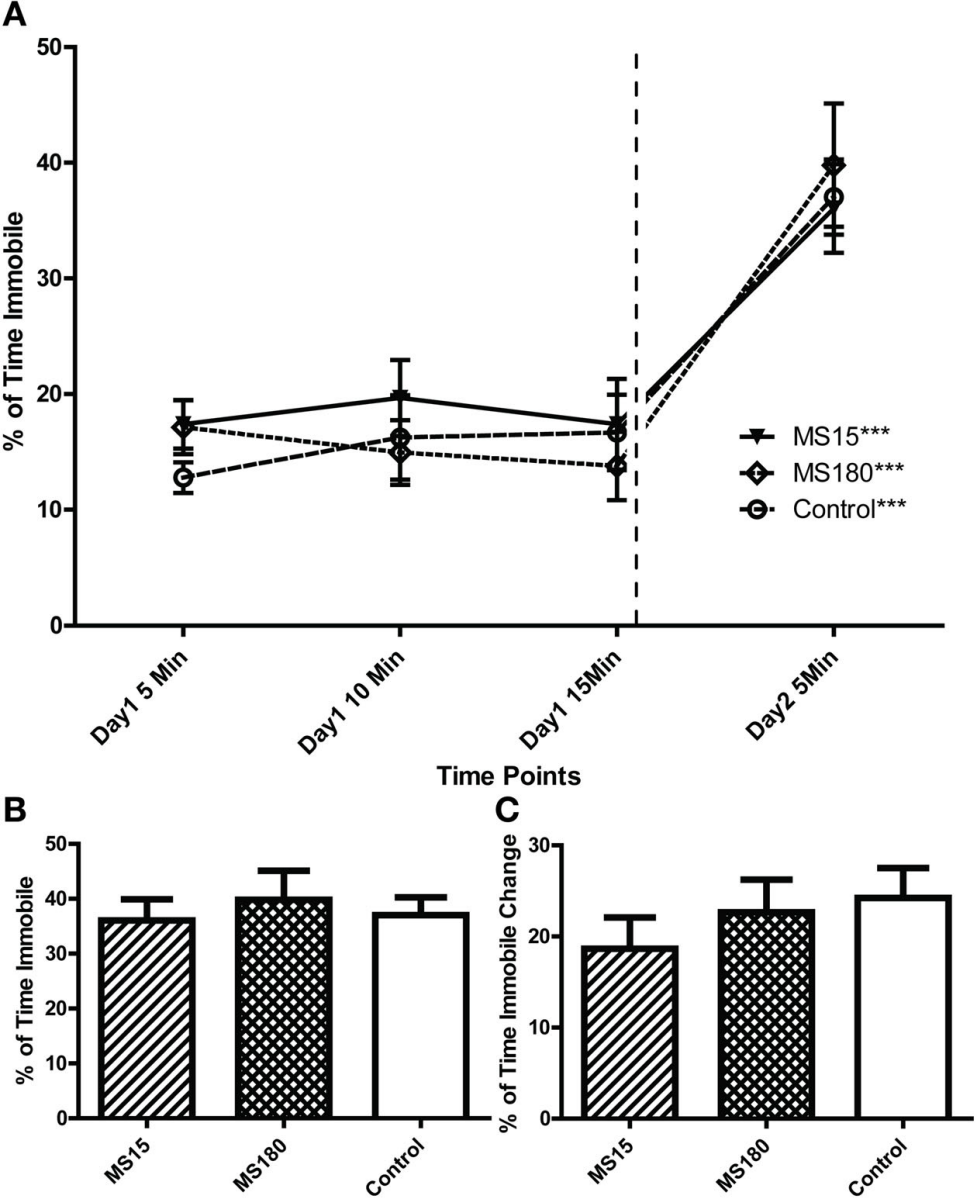
**Early-life experience does not alter WKY depression-like behavior in the forced swim test**. Adult male WKY control rats as well as WKYs that were separated from their dams for 15 min (MS15) or 180 min (MS180) per day during the first 2 weeks of life were examined in the 2-day forced swim test. All groups showed similar immobility levels across Day 1 **(A)** and Day 2 **(A,B)**, and all groups displayed significantly more immobility during the second test session vs. the first **(B,C)** ^***^p < 0.001 for time effect, Day 2 vs. Day 1.

Similar to the first experiment, we analyzed the FST data in two ways. We first compared Day 2 immobility in the three groups: MS15, MS180, and control WKY rats. There was no group difference on immobility [effect of early-life treatment Figure 6; *F*_(2, 42)_ = 0.211, *p* = 0.810]. Next, we performed two-way repeated measures ANOVA to evaluate immobility during the first, middle, and last 5 min of the Day 1 forced swim as well as the Day 2 5-min swim session. This analysis revealed a main effect of time [*F*_(3, 42)_ = 46.26, *p* < 0.001], but no effect of early-life treatment condition and no significant time x treatment interaction. Consistent with the first experiment, all three groups of WKY animals increased immobility on Day 2 vs. Day 1 (*t*-test *p* < 0.001).

#### Open field

**Figure 7:**
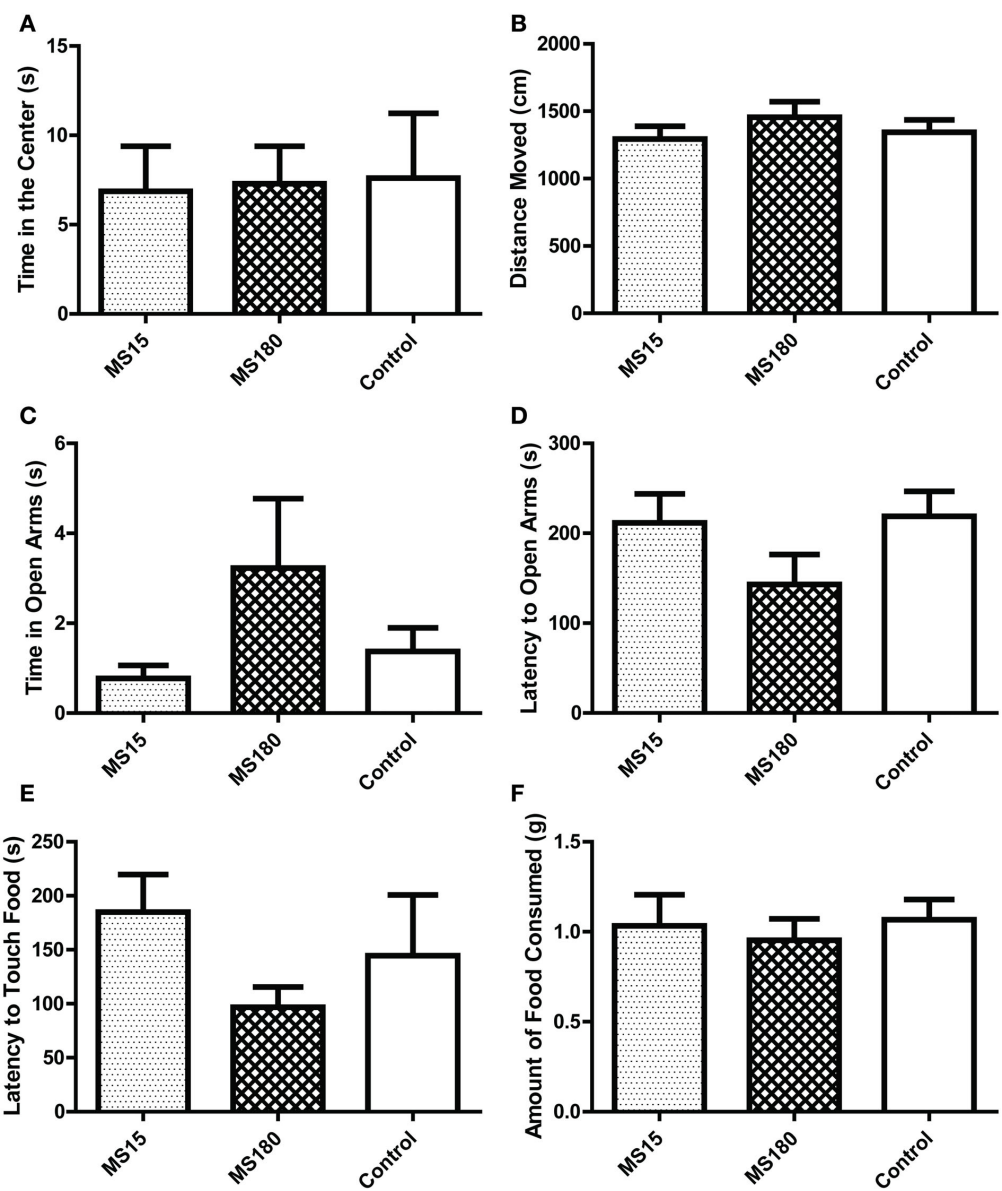
**Early-life experience does not alter WKY anxiety-like behavior in the open field test, elevated plus maze, and novelty-suppressed feeding test**. We evaluated anxiety-like behavior in adult male WKY control rats as well as WKYs that were separated from their dams for 15 min (MS15) or 180 min (MS180) per day during the first 2 weeks of life. All three groups spent similar time in the center of the open field **(A)** and similar locomotion in the open field test **(B)**. Likewise, the three groups performed similarly in the elevated plus maze, spending similar amounts of time in the open arms **(C)** and showing similar latency to enter the open arms **(D)**. The three groups behaved similarly in the novelty-suppressed feeding as well, showing comparable latency to initially touch the food **(E)** and a similar amount of food consumed **(F)**.

As in the first experiment, we examined several measures in the OFT, including time spent in the center (Figure 7A), periphery, and corners of the OF, latency to enter the center (data not shown) and distance of movement during the 5-min test (Figure 7B). There were no group differences between MS15, MS180, and control WKY rats in any of these measures.

#### EPM

We examined several measures in the EPM, including time spent in the open arms (Figure 7C), center square and closed arms of the EPM, latency to enter the open arms (Figure 7D) and mean velocity of movement during the 5-min EPM test (data not shown). There were no group differences between MS15, MS180, and control WKY rats in any of these measures.

#### Novelty-suppressed feeding

In the novelty-suppressed feeding test, we examined the latency for animals to initially touch the food, latency to eat the food, number of food touches and amount of food consumed during the 10-min test. As with the other behavioral tests, there were no group differences between MS15, MS180, and control WKY rats in any of these measures (Figures 7E,F).

## Discussion

The present study demonstrated WKY rats' unique behavioral profile compared to Wistar, SHR, and SD rats. Overall, compared to the other rat strains, WKYs manifested important behavioral differences in response to social and appetitive cues along with a strong propensity to behavioral inhibition in anxiogenic environments and learned helplessness in the FST. In addition, WKY rats also showed unique physiological phenotypes such as heavier adrenal glands relative to the other strains. Our second experiment explored whether WKY's behavioral profile was modifiable by either positive or negative early-life experience (neonatal handling [MS15] or maternal separation [MS180] during the first 2 postnatal weeks). We hypothesized that MS15 would reduce WKY's anxiety- and depressive-like behavior, while MS180 would exacerbate their high anxiety- and depression-like phenotype. Surprisingly, neither early-life manipulation significantly impacted WKY rats' behavior, suggesting a strong influence of their genetic endowment to shaping their behavioral profile that supersedes environmental influences.

### WKYs' unique behavioral profile relative to Wistar, SHR, and SD rats

We observed the most striking and perhaps the most interesting WKY behavioral differences in the FST and the social interaction test. Many previous studies reported WKYs' high levels of FST immobility, although these studies focused only on behavior during the 5-min day 2 swim session of the FST (Paré, 1989; Paré and Redei, 1993; Tejani-Butt et al., 1994, 2003; Armario et al., 1995; Marti and Armario, 1996; Lahmame et al., 1997; Paré and Kluczynski, 1997; Lopez-Rubalcava and Lucki, 2000; Rittenhouse et al., 2002). Our current study shows that WKY rats exhibit high immobility levels on both day 1 and day 2 of the FST, indicating diminished motoric drive in WKYs vs. other strains. Even more interestingly, when evaluating immobility levels across test days, the WKY rats were the only animals that increased immobility level from day 1 to day 2. These findings may be related to an earlier report showing that WKY rats acquire learned helplessness behavior more rapidly than Wistar rats in a shuttlebox escape task following unavoidable shock (Paré, 1994b). The FST is similar to the classic shuttlebox learned helplessness test in that they both use an inescapable stressor to assess behavioral consequences, and both are widely used to test predictive validity of animal models of depression or efficacy of antidepressants (Cryan et al., 2005; Padilla et al., 2011). Indeed, FST immobility has been interpreted as an animal learning that the escape from water is impossible, which suggests it is a model of learned helplessness (Castagné et al., 2011; Stedenfeld et al., 2011). Thus, WKY's behavioral abnormalities in the FST may reflect two functional domains relevant to depression—greater psychomotor retardation as well as enhanced learned helplessness. Furthermore, WKYs' increased immobility over time may be related to the rapidly-acquired and long-lasting avoidance behavior reported in previous WKY studies (Servatius et al., 2008). Future studies will be required to elucidate neural mechanisms underlying these aspects of WKY rats' depression-like behavior.

In the social interaction assessment, WKY rats avoided both the male and female stimulus rats. This is another example of WKYs' high levels of behavioral inhibition and suggests their usefulness as a model of anxiety disorders for which behavioral inhibition is a risk factor (Servatius et al., 2008). Interestingly, WKYs did not avoid novel objects in this paradigm. While WKYs were known to display inhibited behavior in social challenges (Paré, 2000; Ferguson and Cada, 2004), our results showing a specific avoidance of novel stimulus rats but not novel objects demonstrate a specific inhibition to social cues. Accordingly, WKY rats were previously shown to be sensitive to social stress such as social isolation (Malkesman et al., 2006; DaSilva et al., 2011), implicating that they possess a distinct social behavior profile possibly related to their anxiety-like characteristics. WKYs also avoided the female stimulus rat compared to the other strains (particularly compared to the SHRs). SHRs and WKY rats showed equivalent interaction with the male stimulus partner; however, when a female stimulus rat was introduced, SHRs significantly increased their level of interaction while WKY rats remained avoidant. Although the SHR was the only strain that behaved differently when they encountered a male vs. female stimulus rat, WKYs' inhibited behavior toward female partners was particularly unique. As previously suggested, decreased social interaction with females may be interpreted as a sign of anhedonia (Paré, 2000), which is commonly related to depressive disorders in humans.

We were surprised that the WKYs did not show anhedonia in the SPT; they rather preferred sucrose even more than the SD rats. The SPT is frequently used to show increased anhedonia following exposure to chronic stress paradigms (Strekalova et al., 2004; Nestler and Hyman, 2010; Stedenfeld et al., 2011). Our experiments focused on animals at baseline (not following chronic stress), which may explain why we did not observe diminished sucrose-preference in WKY rats. Studies that use SPT for validation of chronic stress typically consider significant decrease in sucrose preference or 50–70% preference cut-off point as a sign of depression-like behavior (Strekalova et al., 2004; Willner, 2005). Although significantly different across strains, sucrose preference higher than 85% may not be biologically meaningful in terms of depressive-like behavior. A specific study should be conducted to test the effects chronic stress on WKY rats in sucrose preference and other behavioral tests.

In terms of baseline ingestive behaviors, it is likely that higher blood pressure levels in SHRs inhibit their thirst via a baroreceptor-mediated mechanism (Stocker et al., 2002), which leads to their significantly lesser water intake as compared to the WKY rats. We also observed that WKY rats exhibit a nearly two-fold increase in sucrose intake (corrected for body weight). This stands in contrast to a previous report demonstrating WKYs' decreased sucrose pellet self-administration compared to the Wistar rats (De la Garza, 2005). However, sucrose pellet self-administration involves instrumental learning and includes an integrated motor response, which complicates interpretation of such findings given the low motor drive of WKY rats (e.g., high level of immobility on day 1 of the FST; generally diminished exploratory activity in novel environments). Sucrose consumption in the home cage, on the other hand, is a more passive and less stressful task; it requires less effort and may be a more accurate indicator of WKYs' hedonic drive to ingest sweetened solutions. Moreover, this observation is reminiscent of the craving for sweets and carbohydrate-rich foods often associated with depression (Fernstrom et al., 1987; Willner et al., 1998).

Our other behavioral results are generally consistent with prior studies documenting WKYs' high levels of depression-like behavior (Paré, 1989; Paré and Redei, 1993; Tejani-Butt et al., 1994, 2003; Armario et al., 1995; Marti and Armario, 1996; Lahmame et al., 1997; Paré and Kluczynski, 1997; Lopez-Rubalcava and Lucki, 2000; Rittenhouse et al., 2002) and anxiety-like behavior (Paré, 1989; Paré and Redei, 1993; Paré and Kluczynski, 1997; Pardon et al., 2002; Ferguson and Gray, 2005; Shepard and Myers, 2008). Characteristics such as high immobility in FST, reduced food consumption in the NSF test, and diminished locomotion in OFT have all been previously reported in WKY rats. Importantly, WKY rats' high FST immobility and low motoric activity in the OFT were present consistently relative to the three other rat strains we examined—SHR, Wistar, and SD. Previous studies have also documented similar differences between WKY rats and other rat strains, including Fischer 344 (F344) and the Lewis rat (Paré, 1989, 1992, 1994a; Armario et al., 1995; Ramos et al., 1997; Will et al., 2003). Interestingly, the WKYs spent a longer time than other strains in the central square of the EPM, which lies at the intersection of the open and closed arms. At least one other group reported this finding earlier, interpreting it as a tendency of WKYs to be more ambivalent or indecisive, which is a trait also observed in clinical depression (Nosek et al., 2008).

WKYs clearly showed diminished movement across multiple behavior tests relative to the other rat strains, indicating a certain degree of behavioral inhibition, psychomotor retardation, and perhaps elevated anxiety. However, WKYs did not exhibit classic anxiety-like behavior for some other anxiety-related measures, such as the time spent in the center of the OF or in the open arms of the EPM, as it has been noted previously (Pardon et al., 2002; Ferguson and Cada, 2004; Getachew et al., 2008). At least one prior study demonstrated that WKY rats exhibit normal locomotor skills in the rotarod test (Ferguson and Cada, 2004). This suggests that their basic motoric ability is normal, but their deficiencies become apparent following stress (e.g., exposure to a novel environment or forced swimming). Alternatively, the fact that these differences depended on a specific test (and the comparison strain) could indicate that WKY rats effectively model certain aspects of anxiety- and depressive-like behavior (such as psychomotor retardation), but not others (such as avoidance of anxiogenic regions like the center of the OF or open arms of the EPM). A further possibility is that a modification of our test conditions (such as lighting intensity) or selection of another anxiety test may have offered a milder anxiogenic stimulus that could have revealed greater inter-strain anxiety-behavior differences.

Throughout our behavioral test battery, we found that SD and Wistar rats exhibited similar behavioral profiles. From a purely behavioral perspective, either strain could therefore serve as a reasonable comparison for WKY rats, especially since they are both outbred rat strains. However, given that the Wistars are derived from the same strain as WKY rats and therefore more similar genetically, they may be a better choice of control than SD, particularly for studies examining possible neurobiological, genetic, and epigenetic factors that contribute to their behavioral differences. Our data indicate that in many instances SHRs may serve as an ideal control for studies of WKY stress-elicited behaviors. For example, in the tests of anxiety-like behavior (EPM and OFT), responses of WKY rats SHRs reveal the most striking contrasts. Specifically, SHRs exhibit a three-fold increase in time spent in the center of the OF and a four-fold increase in the time spent in the open arms of the EPM, while SD and Wistar rats did not differ from WKY rats on these measures. This suggests that SHRs and WKY rats adopt radically different behavioral coping strategies when exposed to anxiogenic environments. Similarly, on the FST, SHRs exhibited no acquisition of behavioral despair, evidenced by no change in the immobility score between swim session on test days 1 and 2, while WKY rats exhibited a two-fold increase in immobility and SD and Wistar rats showed a change of lesser magnitude. One caveat of using SHRs as a comparison is that the line was developed as a model of hypertension, so their unique physiological characteristics may complicate their use as a control for studies with WKYs despite their interesting behavioral profile and close genetic relationship with WKY rats.

### Physiological differences between WKY, Wistar, SHR, and SD rats

In addition to characterizing WKYs' behavioral differences relative to Wistar, SHR, and SD rats, we also examined a subset of physiological measures relevant to stress reactivity—baseline corticosterone levels, body weight and heart and adrenal gland weight. The SHRs had the largest hearts, in concurrence with earlier literature where they were compared to the WKYs (Owens and Schwartz, 1982). As the name suggests, SHRs are chronically hypertensive and this condition accounts for its increased heart size. It was previously shown that SHRs' increased heart weight correlates with greater smooth muscle cell mass and this can be reversed to some degree by anti-hypertensive treatment (Owens and Schwartz, 1982; Owens, 1985). Heart weights were not previously compared across rat strains, and our current data indicate that both SHRs and WKYs have heavier hearts compared to SD or Wistar rats.

We quantified circulating corticosterone levels during the early part of the light cycle, close to the 24-h trough in its levels in its levels in circulation. The absolute levels of circulating corticosterone that we observed are generally consistent with those previously reported in the SHRs, WKY, and SD rats (Gomez et al., 1996; Sterley et al., 2011; Kerman et al., 2012). Our data also indicate significantly higher corticosterone levels in SHRs as compared to WKY, SD, and Wistar rats, which is consistent with a previous report that demonstrated a nearly two-fold increase in circulating corticosterone levels in SHRs as compared to the WKY rats (Sterley et al., 2011). WKY rats did not appear to exhibit higher baseline corticosterone levels compared to SD and Wistar rats, as noted in other studies that assessed corticosterone levels at the 24-h trough (Rittenhouse et al., 2002; De La Garza and Mahoney, 2004). However, WKY rats show higher circulating levels as compared to the Wistar and SD rats during the active cycle around the time of the 24-h corticosterone peak (Gunter et al., 2000; Solberg et al., 2001), but not as compared to the F344 rats (Gunter et al., 2000).

The present study showed that WKYs had the largest adrenal glands compared to the other strains. This is in contrast to previous studies that did not detect significant difference in adrenal weights between WKY rats and SHRs (Lee et al., 1991; Gomez et al., 1996). This may be because we measured adrenal weight in older rats than Lee et al., and Gomez et al. conducted their experiments in females while we used male. Moreover, the animals in our study experienced mild stress of repeated behavioral testing, and chronic stress exposure leads to adrenal cortical hypertrophy and hyperplasia, which result in increased corticosterone secretion and lead to increased adrenal weights in different rat strains, including SHRs, WKY, and F344 rats (Gomez et al., 1996; Ulrich-Lai et al., 2006). Since WKY rats express heightened stress manifestation in peripheral organs, such as stomach ulcer formation (Paré, 1989), it is possible that they would also manifest stress-induced increases in the weights of their adrenal glands following mild stress exposure.

Some of these physiological measures, such as basal corticosterone levels and adrenal weights, have been studied in WKY rats to genetically map these phenotypes (Solberg et al., 2006), in addition to many behavioral measures we have analyzed in this study, including locomotion in OFT (Baum et al., 2006) and immobility in FST (Solberg et al., 2004). Utilizing genetically distinct F334 rats, these previous studies identified multiple quantitative trait loci (QTL) associated with WKYs' distinct physiological and behavioral phenotypes. They also have reported several common loci that may be influencing multiple behavioral traits (Solberg et al., 2004), further confirming that the WKY is a good animal model to study heritable factors that contribute to depression and anxiety in humans. It will be interesting to see if WKYs' distinct social behavior and learned helplessness we report here can also be mapped in QTLs. Moreover, since the SHRs have shown highly contrasting phenotype in our behavioral tests, there is a possibility that distinct behavioral phenotypes of SHRs will also be mapped in the same QTLs as seen in WKY studies.

### Impact of neonatal handling vs. maternal separation on WKYs' behavior

Exposing animals to chronic stress is another approach to model depression in animals besides studying animals with an innate predisposition to high anxiety- and/or depression-like behavior. Combining these approaches by stressing animals with a genetic predisposition for a depression-like phenotype, such as the WKY rat, could offer an even better way to model the disease since clinical depression likely involves both inherited vulnerability factors as well as exposure to various stressors throughout life. Early-life stress manipulations such as prenatal stress, early-life maternal separation and neonatal handling have been shown by numerous groups, including our own, to significantly impact behavior and physiology of rodents (Viau et al., 1993; Bhatnagar and Meaney, 1995; Ladd et al., 2000; Huot et al., 2001; Roth et al., 2009; Clinton et al., 2014). Thus, in the present study, we hypothesized that early-life stress (MS180) would exacerbate WKYs' high anxiety/depression-like behavioral phenotype, but were surprised to find no effects of this manipulation. The results are in accordance with previous studies showing that other early-life manipulations such as cross-fostering and environmental enrichment had little impact on WKY rats' behavior (Paré and Kluczynski, 1997; Howells et al., 2009). Likewise, a report by Sterley et al. found that MS180 did not affect WKYs' performance on the FST or several other measures of anxiety-like behavior (Sterley et al., 2011). We sought to extend previous findings by (a) expanding the behavioral assessment and (b) comparing both MS15 and MS180 conditions side-by-side. We included the MS15 group, in part, because we hypothesized that this manipulation may reduce certain aspects of WKYs' anxiety- and/or depressive-like phenotype. We were therefore surprised again when we found that MS15 had no impact on WKYs' behavioral profile. There might be a floor-effect that hinders us from detecting a possible impact of early-life manipulation on WKYs' behavior considering their already high levels of anxiety-like behavior, especially in the EPM or OFT. It is possible that modifying our behavioral testing procedures, apparatus, or even selection of other anxiety tests would provide a milder anxiogenic stimulus and potentially enable us to detect effects on WKYs' behavioral profile.

Nonetheless, previous WKY studies involving MS15 and environmental enrichment, another positive environmental manipulation shown to decrease rodents' anxiety and stress reactivity, demonstrated similar ineffectiveness on WKYs' anxiety- and depressive-like behavior (Hunziker et al., 1996; Paré and Kluczynski, 1997). Moreover, it has been suggested that the effects of MS15 and environmental enrichment depend on strain, with the impact of manipulations less pronounced in the WKY compared to Wistar (Paré and Kluczynski, 1997). Although the WKY rat is more susceptible to many stressors, it is possible that the WKYs' unique phenotype observed in tests of anxiety- and depressive-like behavior is not easily altered by environmental stimuli that can affect other strains. Recent studies have revealed that maternal separation alters physiological functions of the WKYs, such as GABAA receptor activation (Sterley et al., 2013) or sensitization to angiotensin II-induced hypertension (Loria et al., 2010). These findings imply that early-life experience influences the WKY phenotype, but the effects may predominately elicit physiological or neurobiological changes without substantially altering their anxiety- and depressive-like behavior.

One caveat of the present study is that we did not evaluate the effects of maternal separation on WKY mothers' behavior and interaction with her pups either at baseline or immediately following the daily separation procedure. The quality and quantity of maternal care critically shapes rodent neurodevelopment, emotional behavior, and hormonal stress responsivity (Liu et al., 1997, 2000; Caldji et al., 1998; Francis et al., 1999; Meaney, 2001). Furthermore, maternal separation stress (like other postnatal manipulations) influences maternal care, suggesting that the separation exerts its effect, in part, via subtle behavioral or stress hormone changes in dams when they reunite with litters (Huot et al., 2004; Macri et al., 2011). Previous studies show that WKY mothers interact differently with their pups compared to mothers of other strains, such as Fisher 344 dams (Ahmadiyeh et al., 2004) and SHR dams (Cierpial et al., 1987). Both studies found that WKY mothers showed diminished maternal behavior (showing less licking, grooming, and arched back nursing of with pups) compared to other strains of rat mothers. In the context of the current studies, it would be quite interesting to know (a) whether WKY mothers' baseline maternal behavior is affected by either the MS15 or MS180 separation procedure; and (b) how they may differentially respond once reunited with their pups depending on the length of separation. Future experiments are required to explore these new questions.

There was a noticeable difference in the results of WKY rats' specific behavioral measures between the two experiments (the strain-comparison study vs. the MS15/MS180 study). Specifically, if one compares behavior of WKY rats in the first study (the strain-comparison) with WKY controls in the second study (the maternal separation experiment), rats from the second study exhibited: (a) less FST immobility; (b) increased latency to enter open arms of the EPM; and (c) decreased latency to touch food in the NSF test. These behavioral differences are likely due to the divergent prenatal and early postnatal environments of WKY offspring used in each study. In the first study, WKY rats were born and raised in a commercial breeding facility, which is presumably more stressful than our in-house animal rearing facility where noise, overcrowding, and other external stressors are minimized. In contrast, WKY rats in the second study (the maternal separation experiment) were likely exposed to a greater level of prenatal stress than the other group as they were shipped to our laboratory in the midst of their embryonic development (around embryonic day 15). The offspring were then raised in our animal housing facility, which is likely a less stressful postnatal environment than animals typically experience in a commercial facility. Given the susceptibility of WKY rats to stress (as reviewed above), they may exhibit heightened sensitivity to prenatal and/or early postnatal environments, as both prenatal stress (Vallee et al., 1997; Maccari et al., 2003) and housing environment (Wurbel, 2001; Lewejohann et al., 2006; Macri and Wurbel, 2006) can modify rodent anxiety- and depressive-like behavior. Previous research has also demonstrated that stress exposure at specific developmental time points can result in qualitatively different patterns of physiological and behavioral stress responses (Bingham et al., 2011), so it is not surprising that baseline behavioral differences in the two studies were detected. Our current observations indicate the need to carefully control these early developmental environments. Thus, future studies in which WKY rats are bred in an in-house (non-commercial) animal facility will be required to fully elucidate the impact of maternal separation on these rats. Beyond these issues of prenatal/early postnatal experience, results of animal behavioral tests are very sensitive to a host of environmental conditions, such as noise, lighting, smells, bedding, quality of animal care, air temperature, etc. While we routinely make every effort to standardize these variables in our experiments, it is possible that there were slight differences between the two studies presented here. This highlights the importance of using controls in behavioral studies and unsuitability of using historical controls. Overall, further studies will be required to elucidate potential contribution of prenatal stress exposure and its interaction with early-life experience.

### Perspectives

Our data extend previous observations of heightened depressive- and anxiety-like behaviors within WKY rats and define specific functional domains where WKY rats display their greatest abnormalities relative to other rat strains. In addition to well-known differences in their motoric drive and responses to anxiogenic cues, we show that WKY rats manifest learned helplessness, behavioral inhibition, and diminished social interaction compared to the other strains. WKY rats also appear to be resistant to early-life manipulations known to effectively modify depressive- and anxiety-like behaviors in other rat strains. Therefore, these animals may be particularly useful for studying specific manifestations of clinical depression including: fatigue, learned helplessness, social withdrawal, and associated physiological alterations that contribute to medical co-morbidities. Furthermore, since these rats appear to be resistant to beneficial early-life environmental manipulations (i.e., neonatal handling, cross-fostering, environmental enrichment), they may also be a useful model for the development of personalized anti-depressant treatments.

### Conflict of interest statement

The authors declare that the research was conducted in the absence of any commercial or financial relationships that could be construed as a potential conflict of interest.
